# Supplementary material for: Predicting visual function by interpreting a neuronal wiring diagram
Source: Nature. 2024 Oct 2;634(8032):113–23. doi: 10.1038/s41586-024-07953-5 (PMC11446822; doi:10.1038/s41586-024-07953-5)
Supplement: Supplementary file 6 — CRF and ERF predictions for individual TmY4 and TmY9 cells. Analogous to Supplementary Data 3, but for TmY target types. Shown are the top four monosynaptic pathways, the strongest pathway passing through each of the top ten intermediary types (ranking from Extended Data Fig. 7), and the trisynaptic pathway Tm1–TmY–Dm3–TmY (see the section entitled Prediction of spatial normalization). [file 41586_2024_7953_MOESM6_ESM.zip › DataS4/TmY9qΓèÑ/720575940613539813.pdf]

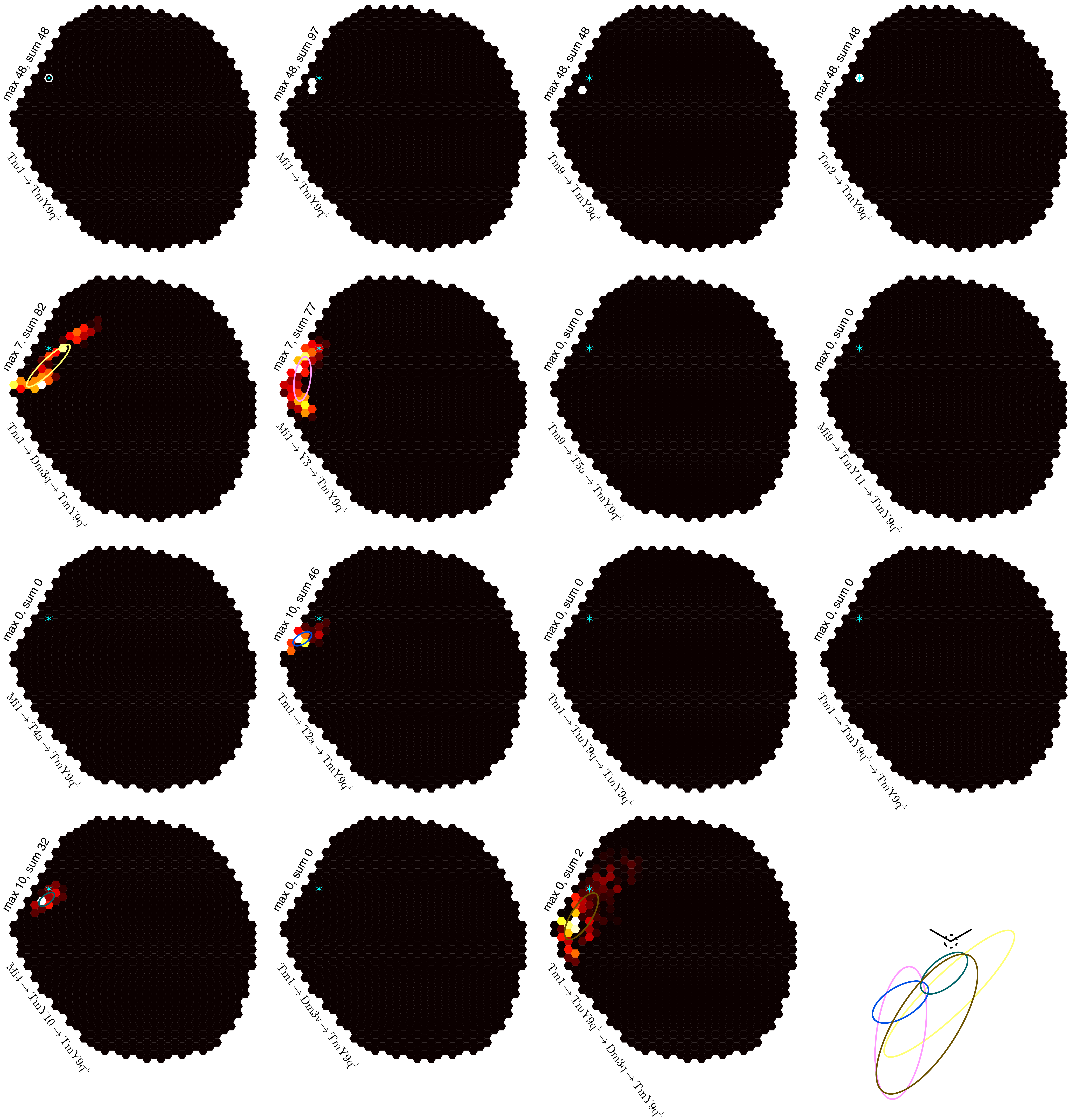

max 48, sum 48
Tm1 → TmY9q⊥
max 48, sum 97
Mi1 → TmY9q⊥
max 48, sum 48
Tm9 → TmY9q⊥
max 48, sum 48
Tm2 → TmY9q⊥
max 7, sum 82
Tm1 → Dm3q → TmY9q⊥
max 7, sum 77
Mi1 → Y3 → TmY9q⊥
max 0, sum 0
Tm9 → T5a → TmY9q⊥
max 0, sum 0
Mi9 → TmY11 → TmY9q⊥
max 0, sum 0
Mi1 → T4a → TmY9q⊥
max 10, sum 46
Tm1 → T2a → TmY9q⊥
max 0, sum 0
Tm1 → TmY9q → TmY9q⊥
max 0, sum 0
Tm1 → TmY9q⊥ → TmY9q⊥
max 10, sum 32
Mi4 → TmY10 → TmY9q⊥
max 0, sum 0
Tm1 → Dm3v → TmY9q⊥
max 0, sum 2
Tm1 → TmY9q⊥ → Dm3q → TmY9q⊥
